# Controlled release delivery of penciclovir via a silicone (MED-4750) polymer: kinetics of drug delivery and efficacy in preventing primary feline herpesvirus infection in culture

**DOI:** 10.1186/1743-422X-11-34

**Published:** 2014-02-22

**Authors:** Samantha L Semenkow, Nicole M Johnson, David J Maggs, Barry J Margulies

**Affiliations:** 1 Towson University Herpes Virus Lab, Department of Biological Sciences, Towson University, Towson, MD 21252, USA; 2 Molecular Biology, Biochemistry, and Bioinformatics Program, Towson University, Towson, MD 21252, USA; 3 Department of Surgical and Radiological Sciences, University of California, Davis, Davis, CA 95616, USA; 4 Department of Pharmacology and Molecular Sciences, The Johns Hopkins University School of Medicine, Baltimore, MD 21205, USA; 5 Department of Pathology, Johns Hopkins University School of Medicine, Baltimore, MD 21205, USA; 6 Department of Biological Sciences, Towson University, Towson MD 21252, USA

**Keywords:** Feline herpesvirus, Penciclovir, Silicone, Implant, Ocular, Controlled release, Herpetic disease

## Abstract

**Background:**

Herpesviruses are ubiquitous pathogens that infect and cause recurrent disease in multiple animal species. Feline herpesvirus-1 (FHV-1), a member of the alphaherpesvirus family, causes respiratory illness and conjunctivitis, and approximately 80% of domestic cats are latently infected. Oral administration of famciclovir or topical application of cidofovir has been shown in masked, placebo-controlled prospective trials to reduce clinical signs and viral shedding in experimentally inoculated cats. However, to the authors’ knowledge, other drugs have not been similarly assessed or were not safe or effective. Likewise, to our knowledge, no drugs have been assessed in a placebo-controlled manner in cats with recrudescent herpetic disease. Controlled-release devices would permit long-term administration of these drugs and enhance compliance.

**Methods:**

We therefore engineered implantable cylindrical devices made from silicone (MED-4750) impregnated with penciclovir, for long-term, steady-state delivery of this drug.

**Results:**

Our data show that these devices release penciclovir with a burst of drug delivery until the tenth day of release, then at an average rate of 5.063 ± 1.704 μg per day through the next 50 days with near zero-order kinetics (in comparison to MED-4750-acyclovir devices, which show the same burst kinetics and average 2.236 ± 0.625 μg/day thereafter). Furthermore, these devices suppress primary infection of FHV-1 in a cell culture system.

**Conclusions:**

The clinical deployment of these silicone-penciclovir devices may allow long-term treatment of FHV-1 infection with a single intervention that could last the life of the host cat.

## Background

Herpesviruses comprise an order of hundreds of pathogens that infect animal species as diverse as oysters to humans [1,2]. A key feature of herpesviral infections is the establishment of latency after primary infection; once an individual is infected, the virus enters a quiescent state and survives throughout the lifetime of the host. This latent state is typically punctuated with sporadic periods of viral reactivation, from which acute-phase or chronic low-grade recrudescent disease can be observed and replicating virus recovered [1,2].

Alphaherpesviruses, a subfamily of this larger order, typically infect epithelial and mucosal surfaces. From there, the viruses retreat into local neurons, where they establish latency. Reactivated virus travels down those same neural axons to infect similar tissues to those that were originally infected, resulting in acute, recurrent, or chronic sequelae at those sites [2-5]. The alphaherpesvirus feline herpesvirus type-1 (FHV-1), serologically detectable in 72-97% of domestic felidae [6-8], is typically transmitted via the respiratory route. The virus usually establishes latency in the trigeminal ganglia, and reactivation can result in keratitis, conjunctivitis, dermatitis, and potentially blindness in affected veterinary patients [9]. Recrudescent disease episodes currently are treated with topically applied ophthalmic solutions or ointments [10-12] or orally administered famciclovir - an orally bioavailable prodrug of penciclovir (PCV) [12-15]; however both of these interventions require consistent administration many times daily, sometimes for protracted periods, which can be associated with poor compliance especially in less tractable cats.

We therefore wished to study a different method of PCV delivery in cats infected with FHV-1. A controlled-release device that contains PCV could potentially be administered once by placement in the subconjunctival space, continuously releasing drug to prevent FHV-1 recurrences over the long term. Therefore, implantable devices, made of silicone MED-4750 (a non-biodegradable polymer that is approved by the FDA for indefinite use in other medical implants) and impregnated with PCV, were constructed for this study. Here we present the basic kinetics of drug release from these devices and their antiviral activity against FHV-1 in culture. We anticipate that these are the first steps in developing better long-term therapy for cats infected with FHV-1. Further, we believe that the highly conserved anatomy and physiology among mammalian eyes and the analogous biological behavior of herpes simplex viruses (HSVs) and FHV-1 in their respective hosts make it likely that data gathered in feline eyes infected with FHV-1 would be predictive of this implant’s behavior once placed subconjunctivally in humans eyes infected with HSV-1.

## Results

### Release kinetics of penciclovir

We have previously demonstrated that the silicone polymers MED-4050 and MED-4750 can be used to deliver the antiherpetic drug acyclovir (ACV) with near zero-order kinetics over at least 60 days [16,17]. To explore the utility of this delivery vehicle for other antiherpetic drugs and to expand upon the range of herpesviruses that might be treated with such devices, we crafted implants of MED-4750 containing PCV. While both ACV and PCV show potent activity against a multitude of alphaherpesviruses [18,19], PCV has greater in vitro antiviral activity against FHV-1 than does ACV [11] and appears to have fewer adverse effects than does ACV in cats [14,20,21], a key requirement for veterinary deployment of any eventual controlled release device.

**Figure 1:**
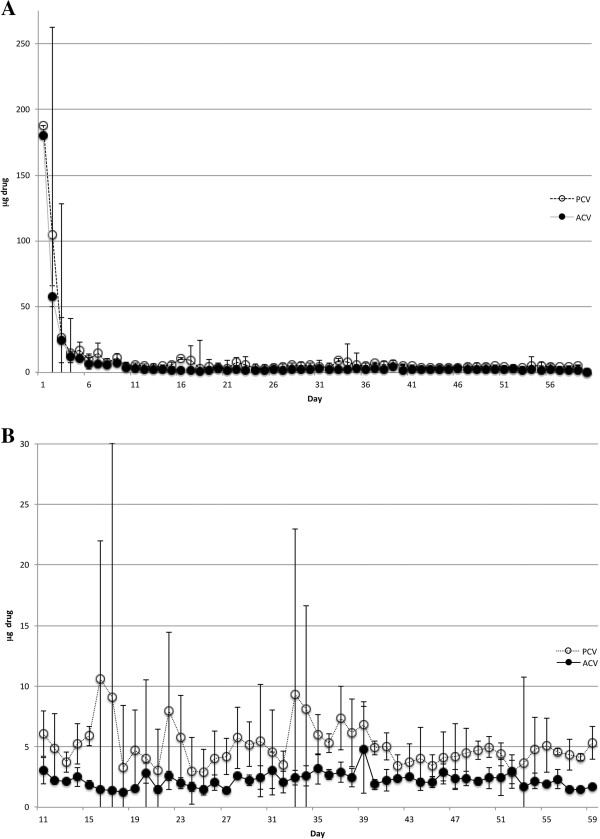
**Release of drug (ACV or PCV) over time from MED-4750 implants maintained at 25°C for 60 days.** Silicone MED-4750 implants containing either ACV or PCV (33% drug load) were incubated in 1 mL PBS that was changed daily. The quantity of each drug from each day’s sample was assayed by HPLC. Each point represents the average and standard deviation of 5 replicates (PCV) or 3 replicates (ACV). **A**. The entire 60-day study. **B**. Study days 11-60.

Implants containing either ACV or PCV (both at 33% drug load) released large amounts of drug during an initial burst phase of approximately 10 days (Figure 1A). These values sometimes reached as high as 180-190 μg of PCV per day. After the initial burst period, though, both drugs were released with near zero-order kinetics (Figure 1B), averaging 5.063 ± 1.704 μg/day (PCV) or 2.236 ± 0.625 μg/day (ACV). These standard deviations are in line with those observed for other silicone-based controlled release devices [22,23]. Therefore, although each set of devices released a different amount of drug per day, both were capable of delivering a constant amount of antiherpetic drug over at least 60 days. It should be carefully noted, though, that the comparison of ACV release to that of PCV is only relevant in the absence of cell culture, since the use of ACV (or valacyclovir) in cats or feline cells in culture is toxic and hampered by poor bioavailability [20,24].

### Protection of CRFK Cells from primary infection with FHV-1

**Figure 2:**
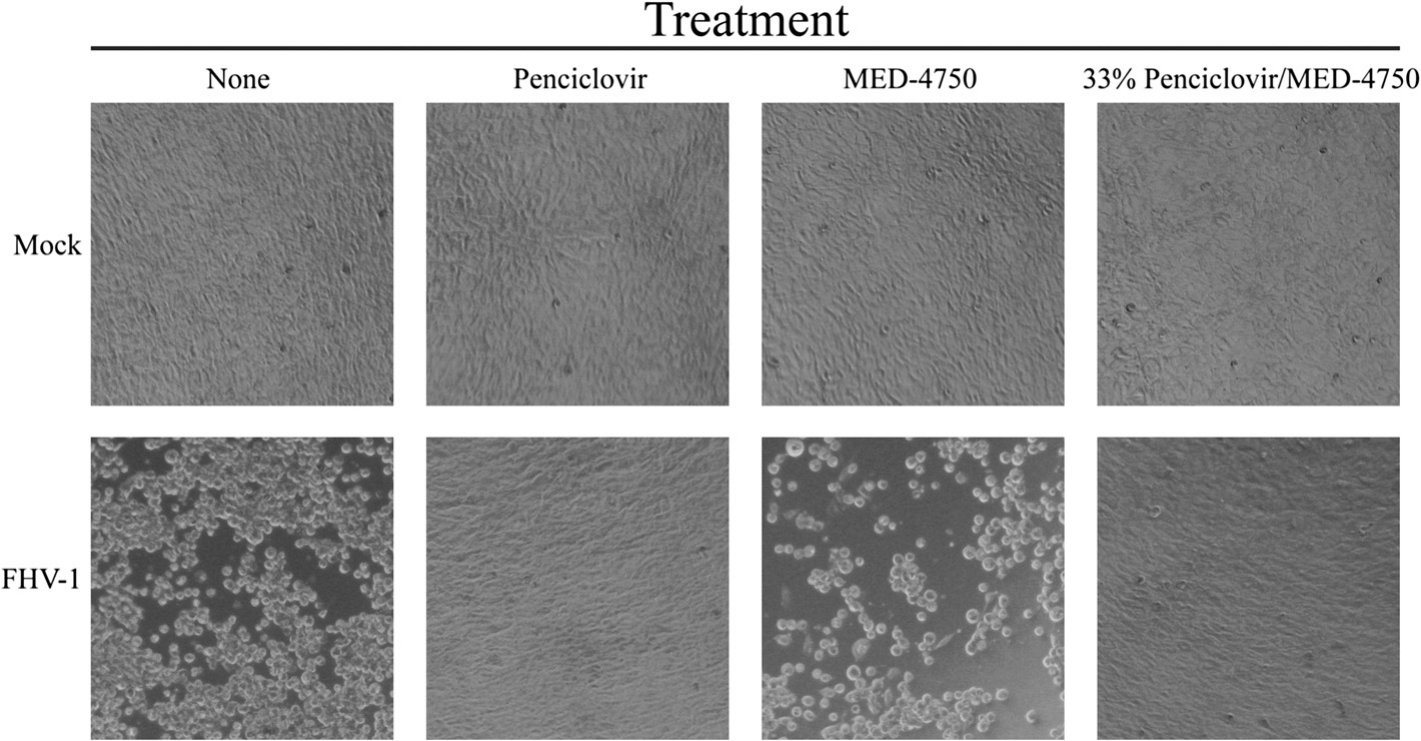
**FHV-1 infection of CRFK cells and protection by PCV-containing silicone implants.** CRFK cells were seeded and treated with nothing (“None”), PCV at 10 μg/mL in PBS (“Penciclovir”), a single 15-mm implant of MED-4750 silicone alone (“MED-4750”), or a single 15-mm implant of MED-4750 containing 33% PCV (“33% Penciclovir/MED-4750”). One day after treatment, FHV-1 (10^2^ pfu/well) was added to cells shown in the bottom row (“FHV-1”) or cells were left uninfected (top row, “Mock”). Photos of these cultures were obtained four days after infection.

We next tested the ability of 33% drug loads of PCV in MED-4750 to inhibit growth of FHV-1 in vitro. CRFK (Crandell Reese feline kidney) cells were grown, treated, and infected as described in the Methods. Photos of these experiments, representative of at least three repetitions of the same experiment yielding identical data, are shown (Figure 2). PCV/MED-4750 implants and PCV in PBS both prevented FHV-1-induced cytopathic effect (CPE; Figure 2, second and fourth columns, respectively). This protection from primary infection was not due to the silicone polymer, as evidenced by large numbers of rounded cells that detached from the tissue culture plate (Figure 2, third column), similar in CPE to cells infected and not treated (Figure 2, first column). No observable adverse effects were seen following treatment with any of these materials in the absence of viral infection (Figure 2, top row).

## Discussion

Herpesvirus infections cause serious acute and recurrent diseases in a significant proportion of the population worldwide and may increase susceptibility to HIV [4,20,24-26]. As a widespread and serious infectious agent for cats, FHV-1 serves as an excellent model for human herpesviruses and their infections [9]. FHV-1 infections are prevalent in the domestic feline population worldwide with up to 97% of cats being seropositive [7], 80% becoming latently infected [27], and up to half of some normal populations shedding virus at any time [28,29]. Infected cats are affected by a variety of cytolytic and immunopathologic ocular and dermatologic syndromes [9], including herpetic stromal keratitis [30], and thereby representing an excellent spontaneous model of human herpetic disease. Typically, alphaherpesviral infections in humans are treated using orally or topically administered drugs such as ACV, the first commercially available antiherpetic nucleoside analogue [9,30,31]. However, ACV has relatively poor in vitro antiviral efficacy versus FHV-1 [11], and is poorly bioavailable [20] and unacceptably toxic [24] in cats. By contrast, PCV is highly efficacious against FHV-1 in vitro [10-12,32], and its oral prodrug famciclovir appears to be safe and efficacious when administered to cats [13,21]. Therefore, while PCV is a suitable drug for treatment of FHV-1-infected cats, current pharmacokinetic data suggest that thrice daily therapy is required [14,21,33]. This is impractical in many clinical situations and associated with considerable expense. Therefore, a long-term, steady-state drug delivery system that could be placed in the subconjunctival space and provide constant FHV-1 suppression would be preferred in cats and might provide an alternative to daily oral maintenance therapy with ACV, famciclovir, or valacyclovir in HSV-infected humans. Such subconjunctival steady-state release systems also are likely to maintain consistent drug levels above the minimum inhibitory concentration and thereby prevent development of drug-resistant viral strains while also improving patient compliance. Similar ocular devices such as the non-biodegradable, ganciclovir-containing intravitreal implant Vitrasert [34,35] are used in humans and bode well for the potential deployment of our subconjunctival silicone-PCV implants in cats. In the present study maximum PCV release during the initial burst phase was 190 μg/day for about 10 days. Assuming a typical adult cat of 3-5 kg body weight, this would result in a systemic dose of 38-65 μg/kg/day, which is more than 1000-fold less than doses that are currently safely administered [14]. Regardless, determination of the in vivo safety and efficacy of these devices placed subconjunctivally in FHV-1infected cats will be an important next step.

Based on the steady-state levels of PCV delivered by these implants in vitro, we expect that a single implant can continuously release drug for 3-17 years. This expectation is centered on the total starting drug quantity encased in a single implant, the initial burst of drug release (Figure 1), the steady-state daily release level in vitro (Figures 1 and 2), and an approximation of expected drug delivery at that rate for about 60% of the drug load; the 60% approximation is derived from calculations for delivery of small molecules from non-biodegradable materials according to Fick and Higuchi [36,37]. Although our previous work with ACV release from the same silicone compound did not suggest a temperature-dependent release rate within the temperature range tested [17], it is important to note that drug release experiments in the present study were conducted at 25°C, whereas in vitro efficacy and cytotoxicity studies were conducted at 37°C. Likewise in vivo use of the implants would be at temperatures above 25°C. Therefore, we project that, given the typical lifespan of domestic cats, a single subconjunctival implant (in essence a single dose), via a single surgical intervention, has the potential to treat a cat for its entire lifetime. Over that lifetime PCV would be expected to reduce the frequency and severity of FHV-1 reactivations and to minimize transmission among cats in multicat settings.

## Conclusion

We report the development of silicone-PCV devices that notably suppress primary FHV-1 infection in cell culture. These devices release a steady-state level of drug (5.063 ± 1.704 μg per day after an initial ten-day burst) for a minimum of 50 days. These implantable devices use an FDA-approved drug and a polymer that has been approved by the FDA as a component of other medical devices; therefore, we envision approval of these devices for medical use once in vivo safety and efficacy are proven.

## Methods

### Implant development

Penciclovir (EMD-Millipore, Billerica, MA and Advance Scientific, Ft. Lauderdale, FL) was combined with silicone MED-4750 (NuSil Silicone Technology, Carpinteria, CA) at a 33% drug load as previously described [16,17], with the following modifications: after milling and mixing, the softened material was placed in a 0.5-inch i.d. polycarbonate cylinder fitted with a matching piston, and extruded through a 2-mm diameter die under pressure in a one-ton arbor press (Harbor Freight Tools, Parkville, MD). The cylinder, piston, and die apparatus were built by Robert Kuta of the Towson University Department of Physics and Geosciences Machine Shop. Implants were cured at room temperature for 7 days, then at 60°C for 24 hours**.** After the curing process, implants were cut to 15-mm lengths. Implants were sterilized by soaking in 40% sodium hypochlorite (household bleach) for one minute at room temperature, transferred through three changes of PBS for 30 seconds each to remove the bleach, then air-dried. Similar methods were used to create a set of silicone-acyclovir (ACV; Advance Scientific) implants containing 33% drug load (w/w) [16].

### Determining in vitro release kinetics

The *in vitro* rate of release of ACV or PCV from the implants was determined by HPLC analysis, as described previously [16]. Implants composed of either 33% penciclovir or 33% acyclovir in MED-4750 were placed into 1.5-mL microcentrifuge tubes with 1.0 mL of phosphate-buffered saline (HyQ® PBS; HyClone, Logan, UT) at pH 7.5 and held at 25°C; this experiment used 5 independent replicates of PCV-containing implants and 3 independent replicates of ACV-containing implants. Every 24 h for 60 days, each implant was moved into a new microcentrifuge tube with fresh PBS. Penciclovir and acyclovir were quantified by high performance liquid chromatography (HPLC). Each sample (50 μL) was prepared for analysis by dilution with 450 μL acetonitrile and analyzed on either an Agilent or Shimadzu system equipped with a Luna HILIC 3 μ, 15 × 100-mm column (Phenomenex, Torrance, CA) under isocratic conditions running 90% acetonitrile/10% formic acid (0.1%) as the mobile phase. Values were calculated against ACV and PCV standards run simultaneously.

### Determining efficacy of implants at preventing viral cytopathic effect in cell culture

Approximately 10^4^ Crandell Reese feline kidney (CRFK) cells were added to a 24-well plate (1 mL per well) at a 1:15 ratio of CRFK cells to medium (Dulbecco’s modified Eagle’s medium (DMEM; Invitrogen, Carlsbad, CA) supplemented with 10% fetal bovine serum (Gemini Bio-Products, West Sacramento, CA) and 1% penicillin-streptomycin-amphotericin B (Mediatech Inc, Herndon, VA). Cells were incubated overnight in 5% CO_2_ at 37°C. The next day, sterile MED-4750 implants containing either 33% penciclovir or no drug were added to two wells for each implant type, one implant per well. Control wells contained 10 μg penciclovir in PBS (two wells) or were untreated (two wells). The cells were incubated in 5% CO_2_ at 37°C for a further 24 hours, at which time one well from each treatment group was inoculated with FHV-1 (10^2^ pfu/well, for an approximate MOI of 0.002). The remaining well of each pair was not inoculated. Photographs were taken four days after infection with a Sony Cyber shot DSC-H2 12 zoom camera attached to an Accu-Scope 3032 microscope with a 10× objective. Final images were compressed and processed with Adobe Photoshop (http://www.adobe.com) and ZereneStacker (zerenesystems.com).

## Competing interests

The work described herein has a related patent pending with the United States Patent and Trademark Office with BJM as an inventor. None of the authors have any competing interests.

## Authors’ contributions

NMJ conducted all the HPLC assays, gathered all the data, and graphed and analyzed those data. SLS executed the infection protection assays and photographed them, and collected samples for the kinetic study. BJM and DJM conceived the studies, supervised the work, and wrote the manuscript equally and collaboratively. BJM also conducted one repetition of the infection protection studies. All authors read and approved the submitted manuscript.
